# Trends in Subcutaneous Cardiac Monitoring Technology

**DOI:** 10.19102/icrm.2018.090703

**Published:** 2018-07-15

**Authors:** Ritsuko Kohno, Teerapat Nantsupawat, David G. Benditt

**Affiliations:** ^1^Cardiac Arrhythmia Center, Cardiovascular Division, University of Minnesota, Minneapolis, MN, USA

**Keywords:** Ambulatory cardiac monitors, insertable cardiac monitors, palpitations, stroke, syncope

## Abstract

Ambulatory cardiac monitoring is a rapidly expanding field and one that is likely to progress beyond electrocardiographic (ECG) and blood pressure recordings. To date, the primary cardiac monitoring focus has been ambulatory ECG (AECG) monitoring. In this setting, AECG monitoring has become a diagnostic tool used daily by physicians of many specialties. In this regard, both wearable and subcutaneous ECG monitoring technologies are now widely available, with the appropriate choice for a given patient being best determined by the frequency with which the patient’s symptom recurrences are expected. In other words, the less frequent the symptomatic events, then the longer the monitoring duration requirement should be. However, multiple factors other than the technology used impact success. For example, wearable AECG systems are only capable of monitoring patients for a period of a few days to several weeks due to limited battery longevity, patient intolerance to cutaneous ECG electrodes, the cumbersome nature of the device, or a combination of these factors. Current-generation insertable cardiac monitors (ICMs), on the other hand, offer three years of monitoring and infrequent skin irritation. Additionally, automatic remote download, a valuable feature in many cases, is only offered by certain wearable technologies, but is an option in all currently available ICMs. This report focuses on the current status of subcutaneous ICMs and their indications and limitations. The goal is to highlight the variety of utility of current ICM technologies and to provide insight into potential future subcutaneous ICM applications.

## Introduction

Ambulatory cardiac monitoring is an expanding field and one that is likely to progress beyond current electrocardiographic (ECG) and blood pressure (BP) recordings to incorporate other potentially valuable clinical diagnostics (eg, hemodynamic assessment, ischemia recognition, metabolic measures, arrhythmia risk assessment). At present, though, most ambulatory cardiac monitoring systems are surface (skin)-mounted and focus on the ECG. However, devices that are inserted into the subcutaneous tissues, or, more rarely, implanted in other organs (eg, pulmonary arterial circulation) have in recent years become increasingly prevalent.

To date, cardiac monitoring has primarily focused on the ambulatory ECG (AECG) domain.^1^ These monitors, available as both wearable and insertable modalities, are now widely used by physicians of many specialties for the evaluation of the causes of symptoms that may be related to cardiac arrhythmias, including palpitations, lightheadedness, physical collapse, and transient or fixed neurological disturbances [eg, transient ischemic attack (TIA), cryptogenic stroke].

In general, the choice of a specific cardiac monitoring technology should be made based on the frequency with which symptom recurrences are expected as well as the nature of symptomatic events.^2^ Thus, the less frequent the symptomatic events, then the longer the monitoring duration requirement generally is. However, factors other than just technology alone impact success. For example, most skin-mounted wearable AECG systems are only capable of monitoring patients from a few days to several weeks (with one month generally being the upper tolerable limit), due to limited battery longevity, patient intolerance to the essential cutaneous ECG electrodes, and/or the cumbersome nature of the devices. In contrast, current-generation insertable cardiac monitors (ICMs) offer recording longevities of up to three years, very low risk of skin irritation or infection, and an almost imperceptible surgical footprint.

Apart from battery longevity, patient compliance determines the duration of effective monitoring. Clearly, ICMs have an advantage, as they accompany the patient at all times and can be downloaded remotely. Nevertheless, even ICM use can be undermined if the patient fails to alert the device when symptoms occur, or is unable or unwilling to permit remote data downloads.

Prompt reporting of actionable ECG findings is one of the most desirable features of cardiac monitoring systems. However, the speed with which important ECG findings are reported to the responsible physician is a significant limitation of many AECG systems. For example, with traditional Holter recorders or first-generation event recorders (eg, ZIO Patch; iRhythm Technologies, San Francisco, CA, USA), the physician may not receive any diagnostic information by way of a report until after the recording period has been completed and the recorder has been returned to the monitoring center. Given the potential for some recording periods to be as long as two to four weeks in duration, the physician may not learn of important ECG events until long after they have occurred. Delayed reporting of actionable cardiac rhythm disturbances may leave patients at unnecessary risk. Again, current ICM systems that have automatic remote transmission of key findings offer a considerable advantage as long as the patient lives in a region that permits remote download (by landline, mobile phone technology, or the Internet) and is sufficiently educated and motivated to use the remote monitoring feature.

This report focuses on the current status of subcutaneous ICMs, including their indications and limitations. In addition, our goal is to provide insight on the potential future of subcutaneous cardiac monitoring.

## Nomenclature and evolution of technology

The initial focus of subcutaneous monitoring efforts was to provide a device capable of “long-term” cardiac rhythm assessment with storage of critical rhythm disturbances and/or symptomatic events (as determined by the patient), with later downloading occurring either in the clinic or by remote telemetry. Further, in order to allow for sufficient time for symptoms to be recognized and electronically marked by the wearer, the recorder was designed to retain key ECG recordings in its memory for a period of time prior to being overwritten. This latter “looping memory” feature provided a crucial diagnostic advantage for subcutaneous systems and, consequently, the term “implantable loop recorder” (ILR), evolved into common usage.

ILRs have changed significantly since their initial introduction into clinical practice by Krahn et al.^3–5^ in the mid-1990s. The earliest prototype device was essentially a modified cardiac pacemaker (Medtronic Inc., Minneapolis, MN, USA) without intracardiac leads, but with sensing electrodes positioned on the device body/header; this latter innovation provided a single-lead subcutaneous ECG recording that could be retained in memory and downloaded at a later date.

In its earliest application, the ILR was primarily used for the management of transient loss of consciousness (TLOC) suspected to be of arrhythmic origin and, subsequently, a substantial body of literature has evolved that supports this application.^6–10^ However, as ILRs have decreased in size and have gained the ability to record and transmit data for longer periods of time (currently, for lengths exceeding three years), their use has expanded into other areas of heart rhythm evaluation. In particular, ICMs are now employed in the diagnosis of infrequent palpitations, for the assessment of treatment efficacy following atrial fibrillation (AF) ablation, and for the evaluation of the cause of cryptogenic stroke.^1,2,6,11,12^

It has become evident that subcutaneous cardiac monitoring has far greater potential utility than ECG detection alone and that, while “looping memory” is important, other features within this evolving technology (eg, remote telemetry download, rhythm-focused diagnostic algorithms, novel physiologic measurements) are comparably notable. Consequently, it is now widely noted that the term “implantable loop recorder” is no longer adequate and that the nomenclature should be superseded by a new broader term, “insertable cardiac monitor,” to reflect both the new monitoring reality and future trends.

## Current insertable cardiac monitoring technology

In recent years, commercially available devices have undergone substantial miniaturization, while at the same time incorporating a number of valuable additional features. The latter includes improved arrhythmia recognition algorithms (particularly for AF), automatic and manual arrhythmia data storage, and remote monitoring capabilities.

**Figure 1:**
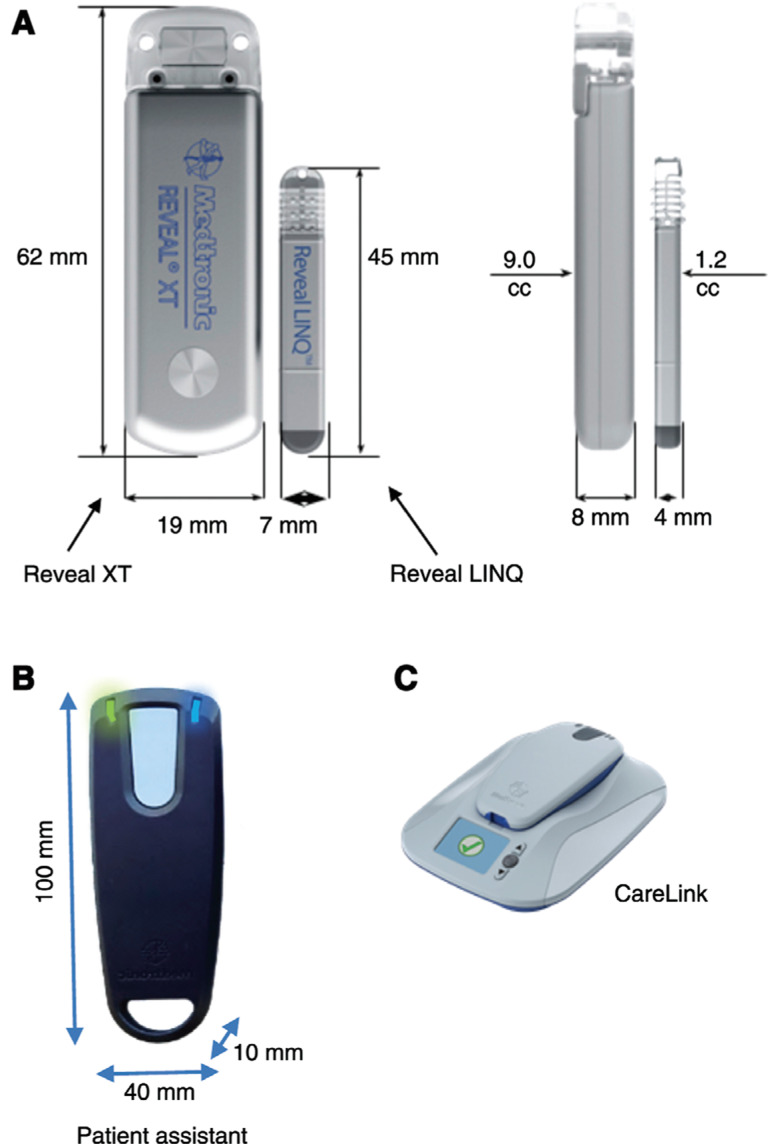
**A:** Reveal^®^ XT™ and Reveal^®^ LINQ™ ICMs (both Medtronic, Minneapolis, MN, USA). The Reveal^®^ XT™ ICM was an early-generation ICM (19 mm × 61 mm × 8 mm in size) and was implanted subcutaneously using a small incision. The Reveal^®^ LINQ™ device is a more recent ICM iteration (7 mm × 45 mm × 4 mm in size) that is approximately 87% smaller than its predecessor. **B:** The Patient Assistant Model 96000, available with the Reveal^®^ LINQ™ Cardiac Monitoring System (Medtronic, Minneapolis, MN, USA). The Patient Assistant feature of the Reveal^®^ LINQ™ device allows patients to mark symptomatic events for further review. **C: **CareLink™ (Medtronic, Minneapolis, MN, USA) for remote monitoring. The CareLink™ Network is used to download patient data from Medtronic implantable cardiac devices.

**Figure 2:**
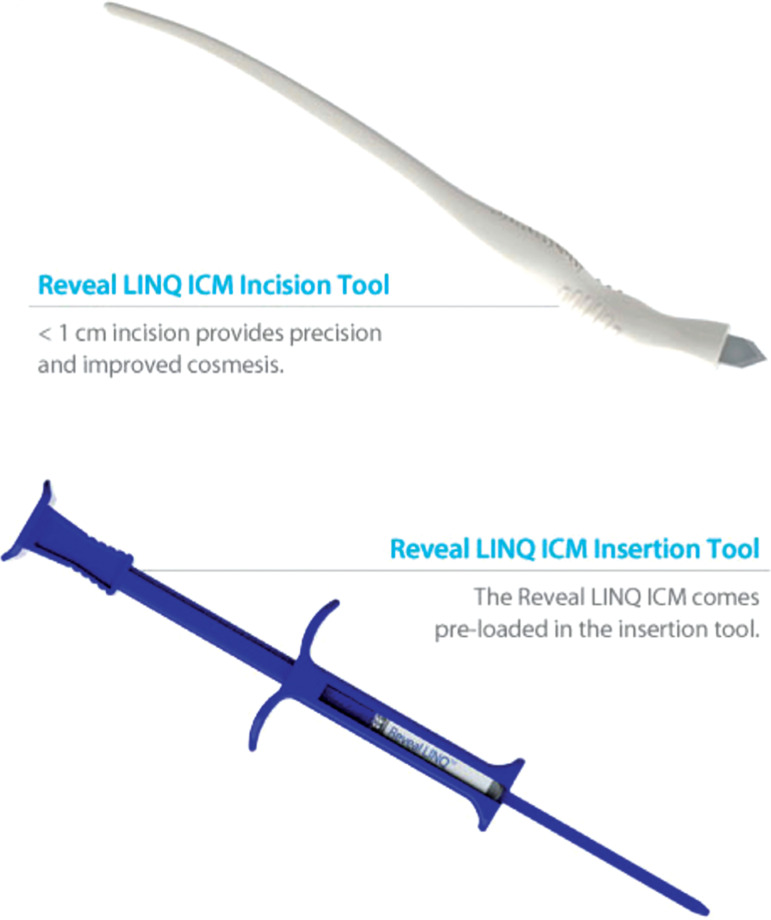
Reveal^®^ LINQ™ insertion blade (top) and preloaded insertion tool (bottom) (both Medtronic, Minneapolis, MN, USA). Once the ICM is injected, the insertion tool is removed and discarded. ICM: insertable cardiac monitor.

The first true ICM (Reveal^®^; Medtronic, Minneapolis, MN, USA) **(Figure 1)** was 19 mm × 61 mm × 8 mm in size and implanted subcutaneously using a small incision. The device was then usually secured to the underlying tissue in order to minimize any potential for migration. The latest iteration of the Reveal^®^ family, the Reveal^®^ LINQ™ (Medtronic, Minneapolis, MN, USA; **Figure 1**), has been reduced to 7 mm × 45 mm × 4 mm in size, making it 87% smaller than its predecessor. The device is provided preloaded in an insertion tool **(Figure 2)** that is used to deliver it subcutaneously through a small puncture incision (< 1 cm) that can then be closed using surgical glue, surgical tape, stitches, or staples as the operator prefers.^11^ As a result, it is now acceptable for the Reveal^®^ LINQ™ (Medtronic, Minneapolis, MN, USA) to be inserted at bedside or in the clinic or emergency department, eliminating the expense of a cardiac catheterization laboratory or operating room.^11,13–15^

**Figure 3:**
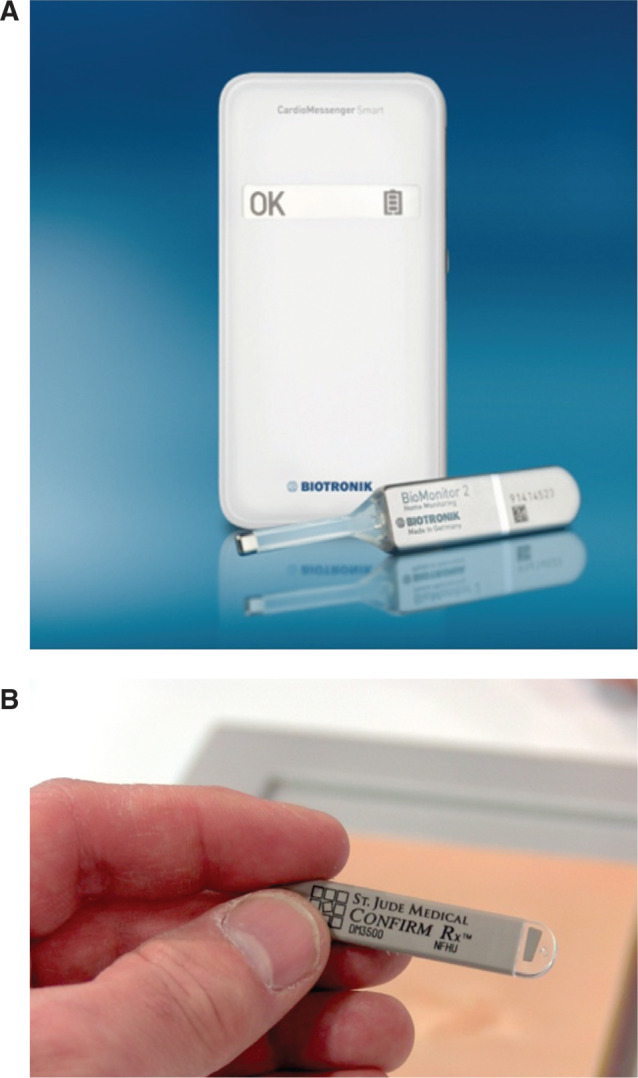
**A:** BioMonitor 2^®^ (Biotronik, Berlin, Germany). **B:** Confirm Rx™ ICM (Abbott Laboratories, Chicago, IL, USA).

In the case of the BioMonitor 2^®^ (Biotronik, Berlin, Germany) **(Figure 3A)**, the housing has the approximate shape and size of a USB flash memory stick. The device has a total length of 8.8 cm with a relatively long antenna reminiscent of the no-longer-available SLEUTH^®^ (Transoma Inc., St Paul, MN, USA). The extended antenna is believed to provide enhanced sensing capability. Further, given its bigger platform in comparison with the Reveal^®^ LINQ™ (Medtronic, Minneapolis, MN, USA) or Confirm Rx™ (Abbott Laboratories, Chicago, IL, USA), the BioMonitor 2^®^ (Biotronik, Berlin, Germany) offers a longer battery longevity of four years. It is also approved for both 1.5-tesla and 3-tesla magnetic resonance environments. Finally, a home monitoring feature automatically collects data from the patient’s device every night, typically while the patient is asleep.

The Confirm Rx™ ICM (Abbott Laboratories, Chicago, IL, USA) **(Figure 3B)** has a relatively small volume (approximately 1.4 cc) with a slim profile. The device is equipped with Bluetooth^®^ wireless technology, allowing patients to connect with it using their own mobile devices.

As alluded to above, current-generation ICMs are capable of both automatic and manual-triggered recording storage and remote monitoring. With regard to remote monitoring, Furukawa et al. reported observations in 47 patients implanted with the Reveal^®^ DX and XT™ ICMs (Medtronic, Minneapolis, MN, USA) and followed using the CareLink^®^ system (Medtronic, Minneapolis, MN, USA) for 20 weeks ± 13 weeks.^12^ The mean time from ICM implantation to the first relevant ECG recording was 28 days ± 49 days, which was estimated to be 71 days ± 17 days less than the identification time acheived using a method of performing three monthly in-office follow-up interrogations and ICM downloads in conjunction with the assumption that the record had not been overwritten in the interim. Furthermore, among a subset of 33 patients queried, all judged the remote monitoring system to be “very easy” (48%) or “easy” (52%) to use and almost all respondents were able to complete the transmission in 10 minutes.^12^ In another report, patients with remote monitoring devices received targeted treatment 187 days earlier on average than when they likely would have received such based on conventional follow-up.^16^ Consequently, it can be said based on these studies that remote monitoring of ICM patients can significantly shorten the time to diagnosis and the time to initiation of the appropriate targeted treatment.

## Overall clinical utility of insertable cardiac monitoring

The “clinical utility” of a diagnostic intervention such as ICM placement may be determined not solely on whether an abnormality is found but, rather, on whether an ICM finding impacts treatment in a positive manner. One example might be the documentation of paroxysmal AF in a patient with cryptogenic stroke that leads to the initiation of oral anticoagulation.

Further, the impact of an ICM observation can be further subdivided into those that were “anticipated” (eg, finding a cause for syncope based on symptom–arrhythmia correlation) or “unanticipated” (eg, finding previously unknown paroxysmal AF in a patient being monitored for some other reason, which triggers the initiation of prophylactic anticoagulation). In either case, one can argue that the patient has received benefit from the intervention. In this regard, Maines et al.^17^ reported positive “anticipated” benefits in 39% of their patients and “unanticipated” benefits in an additional 17%. Similarly, Li et al.^18^ observed “anticipated” benefits in 11.6% and “unanticipated” benefits in an additional 7.4% of 95 ICM patients during a median follow-up period of 414 days.

## Insertable cardiac monitoring limitations

Device miniaturization and simplified implant procedures have reduced barriers to ICM use. Nevertheless, economic issues remaining in some regions may adversely affect uptake, while certain technologic factors also may negatively impact enthusiasm for ICM use among health care providers.

With respect to technology, small ICM size may facilitate implantation but complicate device removal. Years after implantation, very small devices can be difficult to locate, mobilize, and explant. As a consequence, the explant procedure may take longer than the implant procedure, and the explant wound may turn out to be larger than that required for implantation. In the end, explant-related cosmetics could become a concern.

**Figure 4:**
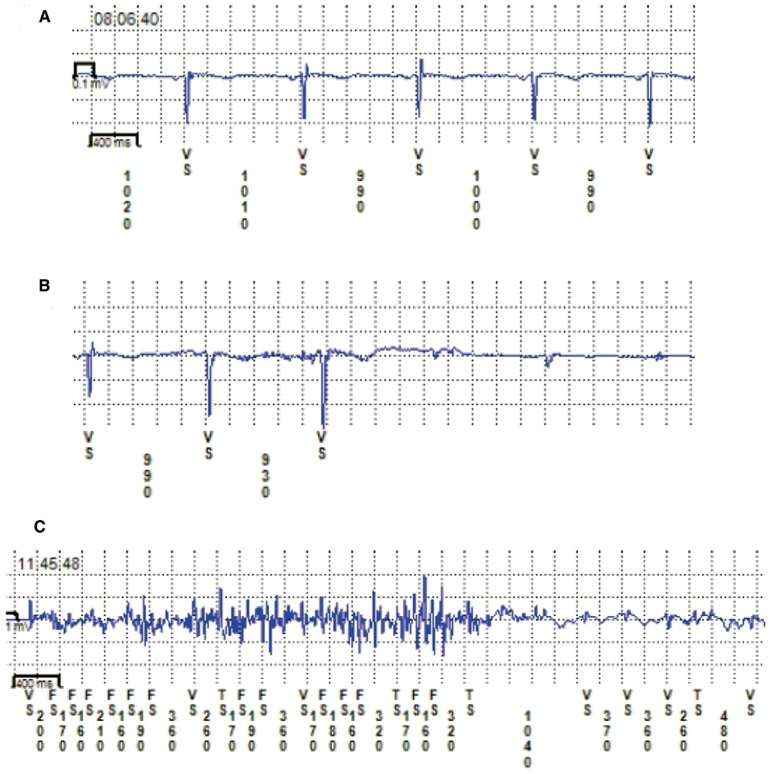
ICM recordings from the Reveal^®^ LINQ™ ICM (Medtronic, Minneapolis, MN, USA) in three patients. **A:** Normal good-quality ventricular electrogram recordings. **B:** Intermittent undersensing of the ventricular signals results in the device incorrectly identifying a “pause” in the patient’s rhythm. This may have been due to movement of the device in the pocket with reduced contact. **C:** Intermittent oversensing of “noise” resulted in incorrect detection of a tachyarrhythmia. Movement of the device in the pocket due to patient manipulation of the ICM could be responsible.

Other issues may also undermine ICM effectiveness. Important among these are ICM “oversensing” and “undersensing” **(Figure 4)**.Inappropriate detections due to physiologic and nonphysiologic circumstances increase episode review time and may reduce diagnostic yield owing to limited episode storage space being available in the device, with possible consequent overwriting of important data by subsequent less crucial events or “noise.” Primary nonphysiologic causes for inappropriate bradycardia and pause detections include undersensing due to loss of electrode contact or a sudden drop in R-wave amplitude (eg, pericardial effusion), or oversensing due to myopotential noise or electromagnetic interference. Physiologic causes for inappropriate detection are primarily related to undersensing of ectopy of ventricular and/or atrial origin. In the latter case, the problem is most likely due to a change in R-wave vector. Several ICM advances—including algorithm enhancements, better tissue contact resulting from small device size and insertion techniques, and longer sensing antennas—may improve automatic detection performance.^19^ In terms of tissue contact, early ICMs required a surgical pocket, albeit a small one. An excessively large pocket undermined tissue contact and signal detection. Even with the newer ICMs that are delivered by an insertion tool, the operator should take care not to swivel the insertion tool, thereby inadvertently creating a pocket larger than necessary and thus potentially adversely altering tissue contact.

Finally, ICMs, unlike wearable loop recorders, require an invasive procedure, which inevitably increases the risks of infection, hematoma, and pain. For example, in the Cryptogenic Stroke and Underlying AF (CRYSTAL AF) trial, which was a controlled study to assess long-term ICM monitoring for the detection of AF after cryptogenic stroke using the Reveal^®^ XT™ (Medtronic, Minneapolis, MN, USA) five of 208 (2.4%) ICMs were removed owing to infection at the insertion site or pocket erosion. Furthermore, the most common adverse events associated with ICM implantation were infection (1.4%), pain (1.4%), and irritation or inflammation (1.9%) at the insertion site.^20^ In this regard, Mittal et al.^11^ analyzed procedure-related adverse events to evaluate the safety profile of the ICM procedure from two separate trials, the Reveal^®^ LINQ™ Usability study (a controlled, nonrandomized multicenter study)^13^ and the Reveal^®^ LINQ™ Registry (a multicenter registry evaluating real-world experiences).^14^ Overall, the combined cohort of 273 patients had an infection rate of 1.5% (n = 4), a procedure-related adverse event rate of 4.0% (n = 11), and a procedure-related serious adverse event rate of 1.1% (n = 3).^11^

In addition to the studies noted above, The Reveal^®^ LINQ™ In-Office 2 study was a randomized trial with the primary objective of comparing the safety of insertion of the Reveal^®^ LINQ™ ICM (Medtronic, Minneapolis, MN, USA) in-office versus in the hospital. This study’s findings indicated that the untoward event rate (a composite of unsuccessful insertion and ICM- or insertion-related complications) was 0.8% (2/244) for in-office and 0.9% (2/227) for in-hospital. Additionally, adverse events occurred during 2.5% (6/244) of in-office insertions and 4.4% (10/227) of in-hospital insertions.^15^ Consequently, at least in the case of the Reveal^®^ LINQ™ ICM (Medtronic, Minneapolis, MN, USA), which is the ICM that has been most thoroughly evaluated to date, both ICM infection rates and overall adverse event rates are low (generally 1%–2% and 2%–4%, respectively)^11,15,20^ and independent of whether the device is placed in the clinic or in the hospital laboratory.

Finally, current ICMs only provide ECG data. Excluding the CardioMEMs™ device (Abbott Laboratories, Chicago, IL, USA), which is primarily used for developing an estimate of left atrial pressure in heart failure patients via placement in the pulmonary arterial system,^21^ current ICMs do not provide hemodynamic monitoring. The addition of hemodynamic assessment capability in future ICMs may help physicians to assess symptoms that patients complain of but which are not associated with overt ECG abnormalities.

## Specific insertable cardiac monitoring clinical applications

### Syncope evaluation

Both observational studies and randomized controlled trials have demonstrated the utility of ICMs in the evaluation of cases of TLOC suspected to be due to syncope of arrhythmic origin but in which the etiology remains unclear after initial evaluation **(Table 1**).

The earliest ICM controlled trial, the Randomized Assessment of Syncope Trial, examined ICM utility in a TLOC, presumed to be syncope, population.^7^ The diagnostic value of ICM (approximately 55%) proved to be superior to that of a wearable external loop recorder (19%) in a prospective study of 60 patients.^7^ These findings are consistent with those of other studies, which generally have shown that patients who underwent the ICM approach experienced higher rates of diagnosis than did those patients who underwent conventional diagnostic approaches.^8–10^ Further, the ICM strategy has proved to be both safe and cost-effective; the mean cost per participant proved to be greater with ICM use, but the cost per diagnosis was lower than that in patients who underwent conventional diagnostic approaches such as tilt or electrophysiological testing.^7–9^

The International Study of Syncope of Uncertain Etiology (ISSUE) has highlighted various aspects of ICM utility in a TLOC population.^22–25^ The initial report (ISSUE-1) summarized findings in 111 patients with presumed syncope, an absence of significant structural heart disease, and a normal ECG who underwent ICM implantation.^22^ Tilt testing was negative in 82 (isolated syncope) and positive in 29 (tilt-positive). Results were similar in the isolated syncope group and in the tilt-positive group: syncope recurred in 28 (34%) and 10 (34%) patients, respectively, while electrocardiographic correlation was found in 24 (23%) and eight (28%) patients. In most patients, the subsequent ICM findings favored a bradyarrhythmic cause of TLOC recurrence. However, only cases with a definitive bradycardia could be identified with certainty by ICM recording; others in which the cause may have been primarily vasodepressor in origin (probably about one-third of recurrences in this study, as only sinus rhythm was recorded during recurrence) could not be diagnosed with certainty.^22^

In ISSUE-3, retrospective analysis suggested that cardiac pacing was primarily of value in individuals in whom ICM showed marked symptomatic bradycardia, but also in whom tilt-table testing did not show vasodepressor susceptibility.^23^ In essence, since reflex syncope typically consists of both cardioinhibitory and vasodepressor aspects, pacing prevention of bradycardia alone may not be adequate to prevent future symptoms unless the vasodepressor component can be shown to be a minor contributor to hypotension.

In regard to the time from implant to diagnosis, in ISSUE-3, during a mean observation period of 15 months ± 11 months, the ICM recorded an event in 187 (37%) of 504 patients, with an estimated probability of 31% at one year, 40% at two years, and 47% at three years.^23^ It is reasonable to assume that, as devices continue to demonstrate greater memory capacity and remote monitoring becomes more widely available, that diagnostic success will increase and the time to diagnosis will progressively shorten.

Among nonrandomized observational reports, the PICTURE registry undertaken in 11 European countries evaluated ICM outcomes in 570 patients with unexplained recurrent syncope or syncope syndrome who were implanted with a Reveal^®^ ICM (Medtronic, Minneapolis, MN, USA).^6^ During follow-up, 218 patients (38%) had symptom recurrences, with ICM recordings identifying a likely cause in 170 of these 218 (77%) patients. Furthermore, 128 (75%) of the 170 patients diagnosed by ICM were deemed to have had cardiac syncope, further emphasizing the importance of ICM findings in establishing a diagnosis and providing direction for therapy.^6^ Finally, in the EASSYAS2 study, strong evidence is provided supporting the synergistic diagnostic use of ICMs with experienced clinicians in specialized “syncope centers.” Such centers are currently uncommon in North America but are becoming increasingly prevalent in Europe.^24^

### Palpitations

While ICM use has focused predominantly on the management of syncope,^7,22–27^ practice guidelines have extended ICM indications to include the investigation of patients with infrequent recurrent palpitations.^28^ The Recurrent Unexplained Palpitations study compared the diagnostic yield and costs of ICM with those of a conventional diagnostic strategy in patients with unexplained palpitations.^29^ Fifty patients with infrequent (one or more episodes per month) and sustained palpitations lasting more than one minute were enrolled. Individuals were randomized either to a conventional diagnostic strategy of 24-hour Holter recording, a four-week period of ambulatory ECG monitoring with an external recorder, and electrophysiological study (n = 24) or to ICM implantation with one-year monitoring (n = 26). In this report, a diagnosis was obtained in five patients with the conventional strategy and in 19 subjects with the monitoring strategy (p < 0.001). Despite the higher initial cost, the cost per diagnosis in the ICM group was lower than that in the conventional strategy group (€3,056 ± €363 versus €6,768 ± €6,672; p = 0.012). It was concluded that ICM use is a safe and more cost-effective diagnostic approach than the use of conventional strategies in subjects without severe heart disease and with infrequent palpitations.^29^

### Cryptogenic stroke

The utility of ICMs for assessing the basis of cryptogenic stroke has recently grown in importance. Clinical trials have demonstrated that a substantial proportion of patients with cryptogenic stroke have previously unrecognized AF that can be detected only by the prolonged monitoring provided by ICM. In this regard, the CRYSTAL AF trial was a controlled study of 441 patients with cryptogenic stroke designed to assess whether long-term monitoring with an ICM—208 patients received Reveal^®^ XT™ devices (Medtronic, Minneapolis, MN, USA)—would be more effective in the detection of AF than conventional management.^20^ By six months, AF had been detected in 8.9% of patients in the ICM group versus in 1.4% of patients in the control group (p < 0.001). By 12 months, AF had been detected in 12.4% of patients in the ICM group versus in 2.0% of patients in the control group (p < 0.001). Similarly, the Stroke Prior to Diagnosis of AF Using Long-term Observation with Implantable Cardiac Monitoring Apparatus Reveal^®^ study was aimed at determining the prevalence of asymptomatic paroxysmal AF in cryptogenic stroke.^30^ Paroxysmal AF was documented in 18 patients (20.7%) during the study period and detected by ICM in 14 patients (16.1%) at a mean of 569 days. Paroxysmal AF was asymptomatic in all cases and occurred in episodes lasting predominantly between one hour and four hours in length. Furthermore, the first event of paroxysmal AF was documented at a mean of 109 days after stroke onset.^30^ Current practice recommendations favor the use of such monitoring in arrhythmia detection after cryptogenic stroke.^20,31–35^

### Postradiofrequency ablation monitoring for atrial fibrillation

ICMs are increasingly becoming used for monitoring for AF recurrence after radiofrequency ablation. Current recommendations favor the use of such monitoring after the ablation of AF, but it is not yet widely employed in practice.^35–37^ In the Assessing Arrhythmia Burden After Catheter Ablation of AF Using an ILR study, 44 patients underwent ICM implantation and conventional monitoring following AF ablation. Subjects were randomized to undergo arrhythmia assessment and management by both ICM and conventional monitoring simultaneously for six months. In the first six months, AF recurred in 18 patients: seven of the 18 cases were detected with conventional monitoring, while all 18 cases were detected with ICM monitoring (p = 0.002). On the other hand, AF was falsely diagnosed frequently by ICM.^35^

### Unexplained falls

Recurrent potentially hazardous falls are a common problem, especially in the elderly and infirm individuals, and are associated with a high hospital admission rate. Falls account for approximately one-third of all adult visits to emergency departments and are accompanied by substantial medical costs (estimates include $30 billion in the United States and more than £2 billion in the United Kingdom).^38^

The Irish Longitudinal Study on Ageing, a population-based study of more than 8,000 community dwelling older adults (aged > 50 years), noted an important overlap among older patients reporting prior faints and those reporting a history of “falls.”^39^ Upon questioning, 16.9% reported prior faints and 4.4% reported faints occurring within the past year. Further, 37.9% of fainters reported having had one or more falls occur within the past year, as compared with 18.3% in nonfainters. Thus, the concern was raised that some falls may in fact have been faints and vice versa. This possibility is further supported by the results of a subsequent ICM study, in which 70 individuals aged older than 50 years with two or more unexplained falls had an ICM placed and were followed for six months to one year (mean: nine months).^38^ In 71% of cases, an arrhythmia was detected. More importantly, however, the study’s findings revealed that, in 20% of cases, the fall symptoms were likely due to treatable arrhythmia [n = 10; 14% received a pacemaker, and four (6%) were treated for supraventricular tachycardia].^39^ Further studies are needed to confirm these observations, given the important clinical problems posed by falls in elderly patients.

## Potential future applications

The diagnostic evaluation of patients with suspected symptomatic arrhythmias is limited by the inability of currently available ICMs to assess the hemodynamic impact of a detected rhythm. Venugopal et al.^40^ reported the use of closely spaced subcutaneous electrodes that were small enough to be incorporated within an ICM to detect pectoral muscle blood flow using electrical bioimpedance changes in a swine model of hemorrhage-induced hypotension. Changes in blood flow-induced pectoral muscle bioimpedance correlated with both a change in mean arterial pressure (p < 0.0001) and in pulse pressure (p < 0.0001). These findings suggest that closely spaced subcutaneous electrodes may prove useful in detecting changes in hemodynamics by identifying changes in local tissue/vascular bioimpedance. Such a device may permit differentiating spontaneous symptoms due to arrhythmia from those caused by transient blood pressure changes such as what occurs in reflex faints.

## Conclusions

Long-term ECG monitoring is essential for the detection of infrequent symptomatic arrhythmias. In this regard, ICM monitoring has proven to be of considerable diagnostic value and is particularly useful when coupled with readily available remote monitoring capabilities. Recent ICM technological developments have enhanced the clinician’s ability to establish the cause of symptoms in patients with suspected arrhythmias in a safe and cost-effective manner.

## Figures and Tables

**Table 1:** Clinical Applications of ICMs

•	In the assessment of unexplained syncope/TLOC
•	For the evaluation of transient symptoms that are possibly occurring due to a cardiac arrhythmia
•	For the monitoring of patients who are at an increased risk of cardiac arrhythmias (eg, those who are postablation or demonstrating drug therapy arrhythmia recurrences)
•	For the identification of the cause of cryptogenic stroke
•	For the evaluation of unexplained falls

ICM: insertable cardiac monitor; TLOC: transient loss of consciousness.
